# Design and Implementation of a National Program of Assessment Model – Integrating Entrustable Professional Activity Assessments in Canadian Specialist Postgraduate Medical Education

**DOI:** 10.5334/pme.956

**Published:** 2024-02-06

**Authors:** Warren J. Cheung, Farhan Bhanji, Wade Gofton, Andrew K. Hall, Jolanta Karpinski, Denyse Richardson, Jason R. Frank, Nancy Dudek

**Affiliations:** 1 Department of Emergency Medicine, University of Ottawa, Ottawa, ON, CA; 2 Royal College of Physicians and Surgeons of Canada, Ottawa, ON, Canada, 1053 Carling Avenue, Rm F660, Ottawa, ON K1Y 4E9, CA; 3 Department of Pediatrics (Critical Care), Faculty of Medicine and Health Sciences, McGill University, Montreal, QC, CA; 4 Royal College of Physicians and Surgeons of Canada, Ottawa, ON, CA; 5 Department of Surgery, Division of Orthopaedic Surgery, University of Ottawa, Ottawa, ON, CA; 6 Department of Medicine, University of Ottawa, Ottawa, ON, CA; 7 Department of Physical Medicine and Rehabilitation, Queen’s University, Kingston, ON, CA; 8 Department of Emergency Medicine, Director, Centre for Innovation in Medical Education, Faculty of Medicine, University of Ottawa, Ottawa, ON, CA; 9 Department of Medicine, Division of Physical Medicine and Rehabilitation, University of Ottawa, Ottawa, ON, CA

## Abstract

Traditional approaches to assessment in health professions education systems, which have generally focused on the summative function of assessment through the development and episodic use of individual high-stakes examinations, may no longer be appropriate in an era of competency based medical education. Contemporary assessment programs should not only ensure collection of high-quality performance data to support robust decision-making on learners’ achievement and competence development but also facilitate the provision of meaningful feedback to learners to support reflective practice and performance improvement. Programmatic assessment is a specific approach to designing assessment systems through the intentional selection and combination of a variety of assessment methods and activities embedded within an educational framework to simultaneously optimize the decision-making and learning function of assessment. It is a core component of competency based medical education and is aligned with the goals of promoting assessment for learning and coaching learners to achieve predefined levels of competence. In Canada, postgraduate specialist medical education has undergone a transformative change to a competency based model centred around entrustable professional activities (EPAs). In this paper, we describe and reflect on the large scale, national implementation of a program of assessment model designed to guide learning and ensure that robust data is collected to support defensible decisions about EPA achievement and progress through training. Reflecting on the design and implications of this assessment system may help others who want to incorporate a competency based approach in their own country.

## Introduction

Programmatic assessment is a core component of competency based medical education (CBME) and is increasingly being adopted into systems of education worldwide [1,2,3]. Central to CBME is the notion that programs have a systematic means to assess the development and achievement of competence of their trainees. This requires clear definitions of desired outcomes and robust assessment systems that accurately identify whether trainees have made sufficient progress to advance, while also facilitating provision of high-quality feedback for learning and supporting reflective practice [4,5]. Programmatic assessment aligns with these goals and is crucial to fulfilling medicine’s social contract to produce competent graduates [6]. Programmatic assessment is a specific approach to designing assessment systems through the intentional selection and combination of a variety of assessment methods and activities embedded within an educational framework to simultaneously optimize the decision-making and learning function of assessment [7,8,9].

Programmatic assessment seeks to address some of the shortcomings of traditional approaches to assessment, which have largely emphasized the summative function of assessment by focusing on the development and use of individual high-stakes assessment tools [10]. Traditional approaches have been criticized because limited snapshots of performance, such as through structured high-stakes examinations, cannot provide sufficient evidence to support robust decision-making regarding learners’ achievement and competence development [11]. Furthermore, when educators rely on periodic assessment activities that are often removed from teaching and learning encounters they miss opportunities to support learner development by providing frequent and meaningful performance feedback, which is needed to enable reflective practice and improvement [10].

By contrast, programmatic assessment shifts away from relying on individual assessment tools; it focuses instead on a “suite” of assessment components that are purposefully and continually collected and analyzed to inform high-stakes decisions (see Table 1) [7]. Programmatic assessment is grounded in the following three fundamental concepts:

1. **Longitudinality**: Programmatic assessment emphasizes longitudinal assessments through training. This supports frequent and ongoing feedback to the trainee to foster reflection and learning, while also generating a continual flow of information to the program that enables tracking of the trainee’s progress over time [7].
2. **Triangulation**: No single assessment is sufficient to support a decision. Rather, assessment information that pertains to the same content is triangulated, and decision reliability draws from the synthesis of multiple data points collected by different assessors, using different methods, over time [12]. Inherent in the concept of triangulation is the need to be deliberate in designing a program of assessment that captures data for each competency domain, and that each domain is informed by a variety of information sources.
3. **Proportionality**: The stakes of an assessment decision should correspond with the richness and trustworthiness of the data used to inform such decisions [8]. High-stakes decisions in postgraduate training have important consequences such as progress and promotion through training. Thus, aggregated quantitative and qualitative data from multiple low-stakes assessments are needed to inform defensible high-stakes decisions. Additionally, because high-stakes decisions occur separately from the individual assessment, each time a learner is observed greater attention can be given to coaching for improvement and to guide learning (i.e., assessment *for* learning).

**Table 1:** Features of programmatic assessment that address criticisms of traditional assessment approaches.


FEATURE	DESCRIPTION

Routine, low-stakes assessment activities are integrated into day-to-day clinical practice	Each assessment encounter serves as a stimulus to provide meaningful longitudinal feedback for development

Intentionally selected assessments and assessment methods are “fit for purpose”.	Intentional selection of assessments and methods supports greater alignment between the intended learning outcomes of the teaching activity and the data collected.

High-stakes decisions are made separately from the individual assessment encounter.	Each assessment encounter is intended to be low stakes, which supports a greater focus on guiding learning (assessment *for* learning).

Decisions are made on the basis of a wide body of evidence that is collected by different assessors, using different methods over time.	The effects of variation due to the specifics of individual cases and contexts, as well as assessor idiosyncrasies, are reduced (provided there is adequate sampling). Additionally, the limitations of one assessment type are countered by the strengths of another.


The advancement in curricular and assessment design that programmatic assessment affords has catalyzed its implementation in an increasing number of medical training programs [7]. While research evidence about the impact of programmatic assessment on learners, teachers, and programs continues to grow [7,13], little has yet been disseminated about the impact of implementation of programmatic assessment on a large scale. In Canada, postgraduate specialist medical education has undergone a transformative change to a competency based model centred around entrustable professional activities (EPAs). In this paper, we describe and reflect on the large scale, national implementation of a program of assessment model designed to guide learning and ensure that robust data is collected to support defensible decisions about EPA achievement and progress through training. In doing so, we make reference to the principles of programmatic assessment as defined by the Ottawa 2020 consensus statement (see Table 2) [8]. These principles represent important and recognizable facets of programmatic assessment. Attention is specifically given to principles 1–8 in this paper. Principles 9–12 are addressed in other papers in this series [14,15].

**Table 2:** Principles of programmatic assessments from the Ottawa 2020 consensus statement for programmatic assessment [8].


1. Every (part of an) assessment is but a data-point

2. Every data-point is optimised for learning by giving meaningful feedback to the learner

3. Pass/fail decisions are not given on a single data-point

4. There is a mix of methods of assessment

5. The method chosen should depend on the educational justification for using that method

6. The distinction between summative and formative is replaced by a continuum of stakes

7. Decision-making on learner progress is proportionally related to the stake

8. Assessment information is triangulated across data-points towards an appropriate framework

9. High-stakes decisions (promotion, graduation) are made in a credible and transparent manner, using a holistic approach

10. Intermediate review is made to discuss and decide with the learner on their progression

11. Learners have recurrent learning meetings with (faculty) mentors/coaches using a self-analysis of all assessment data

12. Programmatic assessment seeks to gradually increase the learner’s agency and accountability for their own learning through the learning being tailored to support individual learning priorities


## Royal College implementation of Entrustable Professional Activities (EPAs)

Competence by Design (CBD) is the model of CBME developed by the Royal College of Physicians and Surgeons of Canada (hereafter referred to as the Royal College) for postgraduate specialist medical training. In CBD, training is organized into four progressive stages. For each stage of training, a set of outcomes have been defined that trainees must achieve before being promoted to the next stage. Each national specialty committee conceptualized and wrote EPAs, with associated CanMEDS milestones, for trainees in its discipline. EPAs are key tasks of the discipline that a trainee can be fully entrusted to perform once they have demonstrated sufficient competence, and milestones represent the component skills required to complete the task [16,17].

Royal College EPAs (RCEPAs) are stage-specific and developmental in nature, meaning that EPAs in later stages are incrementally more complex and build upon EPAs in earlier stages [16]. To progress from one stage to the next, trainees must demonstrate achievement of the EPAs within that stage. Following a comprehensive review of the available data in the trainee’s portfolio, the Competence Committee (CC) makes: a) high-stakes decisions about EPA achievement and b) recommendations to the program about overall trainee progress and promotion through stages of training [14]. A decision about EPA achievement is made when, in the view of the CC and based on multiple observations, a resident can be entrusted to consistently complete the EPA. National guidelines for the context variety and number of successful EPA observations were developed by each of the specialty committees to guide high-stakes CC decisions about EPA achievement in their respective discipline. However, it is up to the CC to determine the type and amount of data required to support their decisions.

**Figure 1:**
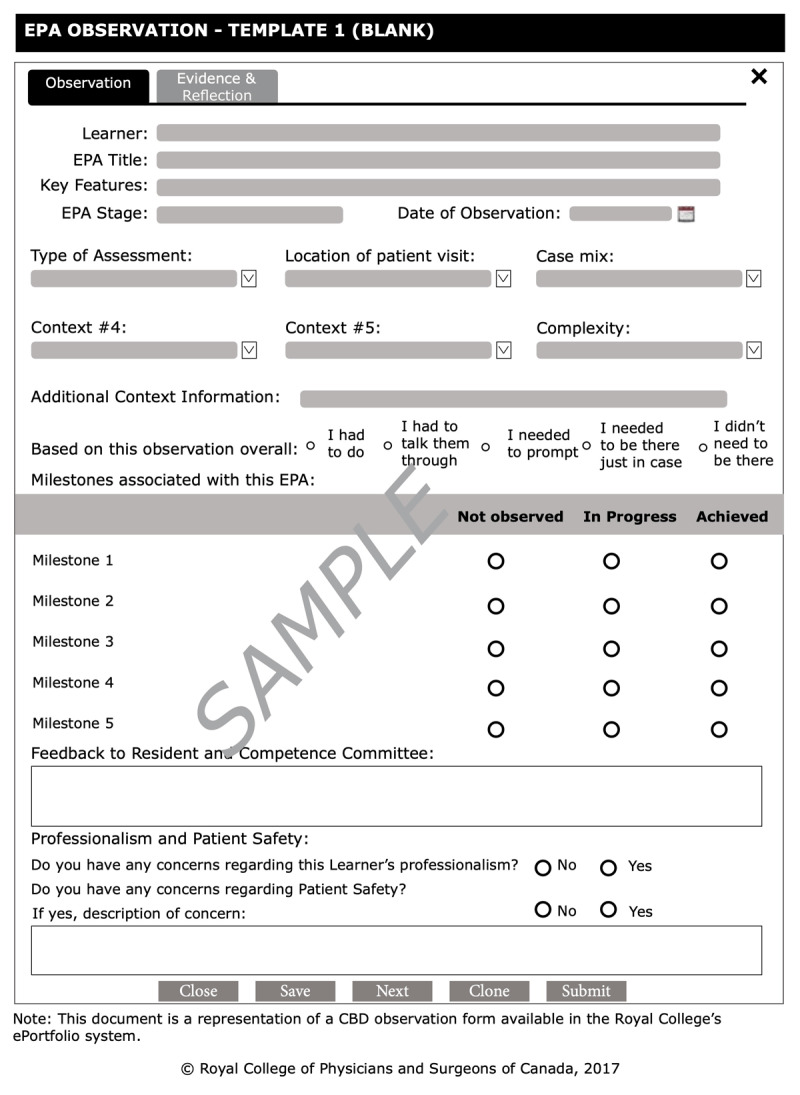
EPA observation form template. *EPA* entrustable professional activity.

EPAs were designed to serve as targets for in-the-moment observation and coaching feedback. Documentation of the observed task was also intended to contribute important workplace-based assessment (WBA) data to inform decisions about EPA achievement by the CC. EPA observations are commonly recorded on an EPA observation form (Figure 1), however programs are given the flexibility to use any WBA tool they find suitable to record the observation. The EPA observation form template was developed as a resource for programs and was designed to facilitate in-the-moment documentation of coaching feedback and judgments of performance. This observation form outlines the key features of the RCEPA and encourages documentation of the specific context in which the task was observed (clinical setting, patient characteristics, case complexity, etc.). Supervisors are asked to provide a single global rating of performance based on the degree of supervision required for that clinical activity. The Royal College strongly encouraged programs to use the O-SCORE rating scale, which has demonstrated strong psychometric characteristics and evidence of validity across a variety of clinical settings [18,19,20,21,22,23]. However, programs were given the local flexibility to use other retrospective supervision scales that indicate a trainee’s level of independence along a developmental arc [24,25]. Milestones for each EPA are also displayed on the form to facilitate the provision of specific and actionable feedback and coaching by “breaking down” the task for supervisors and trainees. A narrative comment section is included to document this feedback.

In the workplace, there is versatility in how EPAs can be observed and assessed through the use of a variety of assessment methods and tools beyond the EPA observation form. EPAs may be directly observed by a clinical supervisor, such as observations of clinical assessments, communication skills, leadership skills, and procedural abilities. They may also be indirectly observed using various methods such as case or chart review, or review of work products such as a consult note. EPAs may also be assessed using data gathered from multisource feedback. Furthermore, qualitative and quantitative data from a variety of WBA tools (not just the EPA observation form) can be aggregated and triangulated to inform CC decisions regarding EPA achievement. Thus, a resident’s ability to perform an EPA can be assessed by supervisors using different methods of observation and recorded using a variety of WBA tools.

The design and implementation of documented EPA observations were intended to align with the principles of programmatic assessment defined in the 2020 Ottawa consensus statement for programmatic assessment (Table 3).

**Table 3:** Documented EPA observation characteristics matched to programmatic assessment principles defined in the 2020 Ottawa consensus statement [8].


EPA OBSERVATION CHARACTERISTIC	PRINCIPLES

Low-stakes, workplace observations are used for in-the-moment feedback and assessment	1–3, 5

Observations are purposefully collected in different contexts, by different assessors, using different methods and tools over time	4, 5, 8

High-stakes decisions about EPA achievement are made separately from the EPA observation by the CC	3, 6, 7

Data from a variety of assessment tools and methods are triangulated to inform high-stakes decisions about EPA achievement	4, 8


Abbreviations: *EPA* entrustable professional activity; *CC competence committee*.

## Beyond EPAs – the program of assessment

The CBD model establishes a national educational framework of stages and EPAs for each discipline that programs must adhere to. However high-stakes decisions about progress through training by the CC are not intended to be solely based on determination of EPA achievement. Rather, programs must also gather assessment information that pertains to competencies and content that are not captured by the EPA framework in order to obtain a comprehensive view of the trainee’s development. Programs are afforded the flexibility to design their own unique “suite” of assessments that integrates a host of both EPA-based and non-EPA based data to inform decisions by the CC about a resident’s progress through training (see Table 2, Principles 6–8).

CBME has been criticized for deconstructing competence into discrete measurable tasks, such as EPAs, at the expense of a more holistic view of trainee development [26,27]. However, EPAs in the CBD context were not designed to be all-encompassing; they simply cannot capture all the requisite knowledge, skills, attitudes and professional expectations of a competent graduate. Rather, they were created to serve as a national framework of training outcomes for programs and trainees. In the design of CBD, an attempt was made to balance the need for practical opportunities for workplace-based observation and assessment of learners by front-line faculty (i.e., EPA framework) [16], while maintaining a holistic overview of the learner through a comprehensive review by the CC of assessment data at all levels of Miller’s pyramid, not just workplace-based data [14,28]. Thus, defensible decisions about trainee progress and promotion in the CBD model requires a complement of assessment information gathered from multiples sources and methods over time that pertain to EPA and non-EPA based content (see Table 2, Principles 4 and 8).

By design, EPAs are essential tasks of a discipline and thus EPA-based assessments target the highest level of Miller’s pyramid by focusing on what a trainee “does” in day-to-day clinical practice [28]. However, diverse assessment methods that address various levels of Miller’s assessment hierarchy are needed to inform defensible high-stakes decisions about trainee progress by the CC [29]. Therefore, in addition to collecting assessment data guided by the national EPA framework, programs must determine what additional non-EPA based data to incorporate in their “suite” of assessments, ensuring that: 1) each assessment type aligns with the purpose of the educational activity for which it was chosen (see Table 2, Principle 5), 2) each assessment generates meaningful feedback to the learner and useful data to inform CC decisions (see Table 2, Principle 2), and 3) data across different assessment activities can be triangulated (see Table 2, Principle 8). A unique feature of the CBD EPA design is the linking of component milestones to and across EPAs, which enables the identification of competencies that are well addressed by EPA observations and those that need other methods of teaching and assessment. Table 4 presents a non-exhaustive list of different assessment types with examples of specific assessment tools that programs may consider integrating into their assessment suite to inform high-stakes progress decisions. To facilitate deliberate selection of their suite of assessments, programs are encouraged to apply the concept of constructive alignment where teaching and learning activities as well as assessment methods are aligned with the intended learning outcomes [30]. This process can be supported through the creation of a curriculum map that explicitly matches: 1) learning activities to stages of training, 2) competencies to learning activities, 3) assessment activities to learning activities, and 4) assessment tools to assessment activities [31].

**Table 4:** Examples of assessment methods and tools by assessment type.


ASSESSMENT TYPE	EXAMPLES

Tests of knowledge	National or local in-training examinations

Progress testing	OSCEs, simulation assessments

Multisource assessments	360 assessment, O-RON [32]

Workplace-based assessments	O-EDShOT [22], OCAT [33], Mini-CEX [34]


Abbreviation: *OSCEs* objective structured clinical examinations.

In summary, the CBD approach provides all programs in the discipline with a national set of EPAs thereby standardizing important clinical learning outcomes and facilitating the implementation of a system of WBA centred around these outcomes. Individual programs are given flexibility in designing a local program of assessment that incorporates both WBAs guided by the national EPA framework as well as non-EPA based assessments to generate a comprehensive picture of the trainee’s development. Royal College accreditation standards and regular accreditation surveys ensure individual programs meet the requirements to develop a holistic program of assessment that addresses the full spectrum of competencies in the discipline and enables defensible progress and promotions decisions.

## Reflections and lessons learned

As with any major change initiative, the Royal College faced early implementation challenges. However, these trials were not universally experienced. For example, disciplines that implemented later benefitted from the past experiences of, and lessons learned by, early implementers. However, even within the same implementation cohort, variability was observed across institutions, disciplines and programs [35]. Thus, efforts to determine and characterize the mediators impacting successful implementation remain an important element of the Royal College’s program evaluation strategy [36]. We also acknowledge that many of these challenges are not unique to CBD implementation and have been previously described in the literature. Some represent “wicked problems” without a single discrete solution that can be applied across each program’s local context – highlighting the complexity of national-scale implementation. Furthermore, while some challenges were anticipated, some represent unintended and unexpected consequences for which an iterative process of evaluation, adaptation and large-scale evolution of CBD is ongoing. Here we reflect on how these challenges uniquely impacted the national implementation of CBD and highlight lessons learned with the hope that they may guide others.

### Challenge 1 – Emphasis on EPA-based assessment data at the exclusion of a program of assessment

With the design and implementation of CBD came a new national framework of EPAs to guide workplace-based assessments. Specialty committees and program directors invested a significant amount of time and effort during their series of CBD workshops to design, refine, and establish their national EPAs [37]. Furthermore, on the front lines, resources were dedicated to on-boarding programs, faculty, and trainees to their new specialty-specific EPAs with the goal of bolstering uptake and engagement. While these efforts were necessary to facilitate the implementation of a new national set of competencies for each discipline, the perceived emphasis on EPAs has had some interrelated and unintended consequences that challenge the principles of programmatic assessment (Table 5).

**Table 5:** Reflections and lessons learned from EPA implementation.


UNINTENDED CONSEQUENCE	PA PRINCIPLES	REFLECTIONS AND LESSONS LEARNED

In some programs, EPAs became the sole target of assessments. The EPA observation form template became the default assessment tool at the exclusion of a “suite” of assessments that capture both EPA and non-EPA based data across different levels of Miller’s pyramid.	4, 8	Program leaders and CCs have identified that relying solely on EPA observation form data results in assessment gaps; they are well positioned to serve as agents to refine the system of assessment [38,39,40,41]. A suite of assessment methods and tools that address multiple levels of Miller’s pyramid and content beyond those captured in the EPA framework are necessary to obtain a holistic view of trainee development and support high-stakes decisions about progress by a CC. During large-scale implementation, while change management efforts will necessarily devote resources to new innovations (e.g., EPA framework), the integration of existing elements (i.e., suite of assessment methods) that will be carried forward must also be supported.

Observation and assessment of EPAs are perceived by trainees as high stakes.	1,2,3,7	The EPA system has been viewed as a set of requirements to progress through the CBD stages of training rather than as a framework to guide opportunities for coaching and growth. National guidelines for the context variety and number of successful EPA observations for achievement have been interpreted as strict requirements, which has promoted a “checklist” mentality around collection of EPA-based assessment data [42,43]. The Royal College has disseminated a technical guide and statement of essential requirements to clarify for programs and trainees that the context variety and number of successful EPA observations should serve as guidance to CCs rather than strict criteria [29,44]. There is an ongoing need to create safe learning environments that promote a growth mindset and enable workplace-based assessments to be perceived as low stakes and positively by learners. Research suggests that a) the trainee’s interaction with the assessor and b) their understanding of the meaning and consequences of the assessment influences their perception of the assessment stakes [43]. The Royal College model of coaching in the moment [15] was developed to help programs and faculty establish positive trainee-assessor interactions that emphasize actionable feedback and optimize the learning function of assessment. National initiatives to clarify the role of EPA observations for programs and residents have been developed and disseminated [45,46]. These initiatives emphasize the learning function of EPA observations, that pass/fail decisions are not made on a single observation, and that many data points collected from various sources are used to inform decisions about EPA achievement and progress.


Abbreviations: *CBD* Competence by Design; *CC* Competence Committee; *EPA* entrustable professional activity; *PA* programmatic assessment.

### Challenge 2 – Terminology impacting the perception of assessment stakes

The design of EPA observation forms by the Royal College included the wide-scale introduction of the O-SCORE rating scale [18,47]. The O-SCORE scale includes anchors that were written using colloquial language to describe the degree of involvement that was necessary by the supervisor for the observed task. The scale has been applied in a variety of clinical contexts and has demonstrated strong psychometric characteristics including reliable scores and the ability to discriminate training level [19,20,22,23,48]. The O-SCORE, and other similar scales, were initially described as *entrustability scales* [49]. What the Royal College did not anticipate was that front-line faculty would perceive the rating they provided on a single EPA observation form to be a high-stakes judgment of whether or not the trainee could be fully entrusted to perform the task in the future (a decision reserved for the CC based on triangulation of multiple data points collected in different contexts over time; see Table 2, Principles 7 and 8). The wider medical education community raised concerns that the term *entrustability scale* was inadvertently and erroneously conveying a message to faculty that the rating of performance they documented on the EPA observation form was a determination of the resident’s future entrustment [50], thus raising the perceived stakes of these assessment and placing undue burden and responsibility on front-line faculty (see Table 2, Principles 3 and 7) [24,51].

#### Reflections and lessons learned

In an effort to dispel the misconception surrounding the stakes of faculty judgments of EPA performance, the Royal College is considering transitioning toward the term *retrospective supervision scale* [24], to remove the term ‘entrustability’ and any high-stakes connotations it might hold. Additionally, ongoing resources are being developed and disseminated for faculty development to help assessors better understand the learning goal of their assessments (low stakes, focused on feedback and growth based on the observed encounter). The educational impact of the assessments is derived largely from the narrative feedback to learners, and so ongoing faculty development strategies to improve the quality of narrative comments documented on assessments has been a focus as the Royal College continues to enhance CBD implementation.

### Challenge 3 – Off-service rotations

Assessing trainees during off-service rotations can be challenging using a system of discipline-specific EPAs – like trying to fit a square peg in a round hole – for a few reasons. First, because the assessors are not in the same specialty as the trainee, they may not be familiar with the standard for competent independent practice in the trainee’s specialty. Second, EPAs are discipline-specific tasks and may not be observable in a different clinical environment. While some specialty committees were deliberate in designing EPAs that could be observed and assessed on off-service rotations, this was variably considered across disciplines. Third, the goal of an off-service rotation may not be to acquire the ability to perform a task independently (i.e., an EPA); it may instead be knowledge acquisition or skill development. As a result, asking off-service faculty to complete assessments of EPAs may not be relevant to the intended learning outcomes and may not facilitate documentation of useful information to the degree that many programs feel is necessary. Fourth, orienting faculty to what they should teach and assess for off-service trainees rotating from a variety of different specialties is challenging.

#### Reflections and lessons learned

A clear understanding of why trainees are going to a particular off-service rotation should be established by the home program and the assessment tools used should reflect these goals (see Table 2, Principle 5). Using other types of assessment tools (besides EPA observation forms) to capture the details of how trainees are developing during the off-service rotation can provide the CC with more meaningful data. It also helps to ensure that trainees are being exposed to appropriate training experiences during these rotations. Providing off-service faculty with an orientation to the goals of these rotations, what should be assessed and the types of assessment tools to be used can improve the quality of performance data gathered during these rotations. However, it is recognized that this will not always be possible because of the significant number of faculty who may be involved. As such, making the assessment tool as user friendly as possible can help.

### Challenge 4 – Resident burden of assessment

Many programs have set up their assessment systems such that the trainee is solely responsible for driving EPA observations. While there is certainly value in having trainees direct their own learning, exclusive reliance on trainee-driven assessments has had some unintended consequences. These include an increased burden of work for trainees [42], negative impacts on resident wellness [52], limitations with respect to which faculty complete assessments, restrictions of assessments to those where the trainee demonstrated independent performance [53], and a smaller number of documented observations overall.

#### Reflections and lessons learned

Initiation of assessment activities should be a responsibility shared by both faculty and trainees. While trainees may be most familiar with their areas of strength and what domains of practice they require more observation and coaching, faculty may be more attuned to pick up on deficiencies that have not yet been identified by the trainee. Thus, assessments should be triggered by both faculty and trainees to ensure that the burden of this work is shared, a wide variety of faculty and range of observers are involved, and trainees receive routine and documented feedback on clinical performance as they progress toward independence. Mapping EPAs to particular rotations can help faculty become more familiar with a subset of tasks that they will routinely observe and assess (see Table 2, Principle 5) and decrease the effort they need to invest in trainee assessment. As well, programs that have set an expectation with regard to the number of documented observations that should be triggered per week and by whom have had more success in addressing the above challenges.

### Challenge 5 – Real-world implementation of EPAs

While program directors and specialty committees made every effort to design EPAs to reflect the key tasks of their discipline, it became apparent early in implementation that some EPAs were not congruent with practical daily workflow (e.g., direct observation of tasks by faculty who are on home-call). Understandably, this has had an impact on the acceptability, perceived validity, and educational impact of such EPA observations (see Table 2, Principles 2 and 5) [54,55,56].

#### Reflections and lessons learned

Margaret Atwood has said, “If I waited for perfection, I would never write a word [57].” As with any major change initiative, the Royal College aimed to iterate and refine, using a process of continuous quality improvement, the national set of EPAs at the specialty committee level. While some disciplines that were among the first to launch CBD have made refinements to their original EPAs, other disciplines are only now beginning the revision process. The Royal College recognized that the capacity to support EPA revisions at the specialty committee level will be impacted by resource constraints. Educators intending to implement large-scale programs of assessment should consider piloting at a smaller scale before full implementation. Piloting can help to identify unanticipated challenges and areas of increased resource needs, establish infrastructure for ongoing development, and ensure sufficient capacity to facilitate ongoing quality improvements [58].

## Conclusion

The implementation of a program of assessment model designed to guide learning while ensuring collection of robust data to support defensible decisions about EPA achievement and progress through training on a national level across multiple disciplines is a complex process and a major change initiative. We have described the CBD model of programmatic assessment that integrates a WBA system centred around EPAs and have reflected on the challenges along this journey. It is our hope that this paper offers valuable insights for other educators who are intending to embark on a large-scale transformation of their system of assessment.

## Disclaimer

The views and opinions expressed in this article are those of the authors and do not necessarily reflect the official policy or position of the Royal College of Physicians and Surgeons of Canada (“Royal College”). Information in this article about Competence by Design (“CBD”), its implementation and related policies and procedures do not necessarily reflect the current standards, policies and practices of the Royal College. Please refer to the Royal College website for current information.
